# Outsourcing Medical Data Analyses: Can Technology Overcome Legal, Privacy, and Confidentiality Issues?

**DOI:** 10.2196/jmir.2471

**Published:** 2013-12-16

**Authors:** Bostjan Brumen, Marjan Heričko, Andrej Sevčnikar, Jernej Završnik, Marko Hölbl

**Affiliations:** ^1^Institute of InformaticsFaculty of Electrical Engineering and Computer ScienceUniversity of MariborMariborSlovenia; ^2^Health Care Center MariborMariborSlovenia

**Keywords:** confidentiality, patient data privacy, data protection, medical decision making, computer-assisted, data analysis

## Abstract

**Background:**

Medical data are gold mines for deriving the knowledge that could change the course of a single patient’s life or even the health of the entire population. A data analyst needs to have full access to relevant data, but full access may be denied by privacy and confidentiality of medical data legal regulations, especially when the data analyst is not affiliated with the data owner.

**Objective:**

Our first objective was to analyze the privacy and confidentiality issues and the associated regulations pertaining to medical data, and to identify technologies to properly address these issues. Our second objective was to develop a procedure to protect medical data in such a way that the outsourced analyst would be capable of doing analyses on protected data and the results would be comparable, if not the same, as if they had been done on the original data. Specifically, our hypothesis was there would not be a difference between the outsourced decision trees built on encrypted data and the ones built on original data.

**Methods:**

Using formal definitions, we developed an algorithm to protect medical data for outsourced analyses. The algorithm was applied to publicly available datasets (N=30) from the medical and life sciences fields. The analyses were performed on the original and the protected datasets and the results of the analyses were compared. Bootstrapped paired *t* tests for 2 dependent samples were used to test whether the mean differences in size, number of leaves, and the accuracy of the original and the encrypted decision trees were significantly different.

**Results:**

The decision trees built on encrypted data were virtually the same as those built on original data. Out of 30 datasets, 100% of the trees had identical accuracy. The size of a tree and the number of leaves was different only once (1/30, 3%, *P*=.19).

**Conclusions:**

The proposed algorithm encrypts a file with plain text medical data into an encrypted file with the data protected in such a way that external data analyses are still possible. The results show that the results of analyses on original and on protected data are identical or comparably similar. The approach addresses the privacy and confidentiality issues that arise with medical data and is adherent to strict legal rules in the United States and Europe regarding the processing of the medical data.

## Introduction

### Background

Medical data are gold mines for deriving knowledge. Hiding within those mounds of data is knowledge that could change the life of a single patient, or sometimes change the health of an entire population [1]. Medical doctors—field experts—use the data collected from various sources on a daily basis for treating patients. Many data from examinations and laboratory tests require further analyses, which can be very time consuming and require experts to conduct them. In fact, the amount of data produced by medical electronic equipment is enormous and continues to grow at a very fast rate—the amount of data doubles in approximately 15 months [1-3]. The large volumes of data make human-driven analyses impossible. Machine support and intelligent data analyses are definitely required. Medical experts and other employees of a health care service provider generally do not possess in-house expertise for doing automatic data analyses. There is also a distinction between deriving knowledge from Web pages, blogs, social media systems, etc, and from the closed systems typically present in medical environments. The former is mostly used for branding purposes (advertising, marketing, and content delivery) [4] and the latter to support doctor’s decision making. Sometimes information technology (IT)-related resources in a health care system, including hardware and software, may not be adequate or even available for the data analyses aimed at knowledge discovery. The obvious choice is to have a third party conduct the analyses.

Here, privacy and confidentiality issues arise together with legal obligations. Respect for privacy has been a part of the medical profession since ancient times. “Whatever I see or hear in the lives of my patients, whether in connection with my professional practice or not, which ought not to be spoken of outside, I will keep secret, as considering all such things to be private...” is the text from an oath attributed to Hippocrates referring to confidentiality [5]. Privacy and confidentiality are very important contemporary issues, especially in the Western world, and are not limited to the medical field.

Privacy has (re)emerged as an important issue since the emergence of social media, as noted by Mark Zukerberg, the founder of the most-used social network, Facebook [6], and Facebook’s chief operation officer, Sheryl Sandberg. They observed that privacy controls were centered at Facebook’s core at all times [7,8]. Indeed, privacy needs to be considered seriously from a technological point of view when designing applications and solutions. In Canada, the Ontario Privacy Commissioner, Ann Cavoukian, has developed a Privacy by Design (PbD) framework [9-11] which emphasizes the need to adopt a proactive rather than a reactive compliance approach to the protection of privacy.

The laws of most developed countries impose obligations to respect informational privacy (eg, confidentiality, anonymity, secrecy, and data security), physical privacy (eg, modesty and bodily integrity), associational privacy (eg, intimate sharing of death, illness, and recovery), proprietary privacy (eg, self-ownership and control over personal identifiers, genetic data, and body tissues), and decisional privacy (eg, autonomy and choice in medical decision making) [12,13]. In this paper, however, we address the first type of privacy: informational privacy.

Informational privacy is usually violated by a data breach, which can result from theft, intentional or accidental unauthorized access to data, acts of revenge by unsatisfied employees, or by the accidental loss of media or devices that bear data.

Despite the regulations in place, the stories of privacy and confidentiality breaches are still frequent. Major hospitals and health-related institutions, most notably in the United States but also elsewhere in the world, have experienced highly publicized data breaches—more than 770 breaches have occurred since 2005 in the United States alone [14]. Anciaux et al [15] observed that traditional electronic health records (EHR) have no security guarantee outside the health care service domain and pervasive health, a new concept based on latest developments, requires implementable principles for privacy and trustworthiness [16]. Van der Linden et al [17] noticed that before the virtual lifelong patient record can become reality, more clarity has to be provided on the legal and computational frameworks that protect confidentiality.

Can technology help and how can it help? First, let us take a closer look at definitions of privacy and confidentiality and how they are reflected in laws and rules.

### Privacy and Confidentiality

Daniel Solove [18] has stated: “Privacy is a concept in disarray. Nobody can articulate what it means.” But one must note that privacy and confidentiality do share at least some common grounds among the philosophers and jurists, and many technologies exist that address privacy and confidentiality.

In Ancient Greek civilization, there existed 2 interdependent and sometimes conflicting areas: the public area of politics and political activity, the *polis*, and the private area of the family, the *oikos* [19,20]. These areas were reflected in classic dramas (eg, in Sophocles’ *Antigone* and *Oedipus Rex*), and the new order of the polis, despite its weaknesses, reigned supreme at the end of the dramas [21].

More systematic discussion of the concept of privacy began with an article by Samuel Warren and Louis Brandeis titled “The Right to Privacy” [22]. Citing “political, social, and economic changes” and a recognition of “the right to be let alone,” they argued that existing laws afforded a way to protect the privacy of the individual, and they sought to explain the nature and extent of that protection. Focusing in large part on the press and publicity allowed by recent inventions, such as photography and newspapers, but referring to violations in other contexts as well, they emphasized the invasion of privacy brought about by public dissemination of details relating to a person’s private life. Warren and Brandeis felt a variety of existing cases could be protected under a more general right to privacy which would protect the extent to which one’s thoughts, sentiments, and emotions could be shared with others. They were not attempting to protect the items produced or intellectual property, but rather the peace of mind attained with such protection; they said the right to privacy was based on a principle of “inviolate personality” which was part of a general right of immunity of the person: “the right to one’s personality” [22]. Thus, Warren and Brandeis laid the legal foundation for a concept of privacy that has come to be known as control over information about oneself [23].

In an attempt to systematize and more clearly describe and define the new right of privacy upheld in tort law, William Prosser [24] wrote in 1960 about 4 different interests in privacy, or privacy rights:

1. Intrusion upon a person’s seclusion or solitude, or into his private affairs;
2. Public disclosure of embarrassing private facts about an individual;
3. Publicity placing one in a false light in the public eye; and
4. Appropriation of one’s likeness for the advantage of another [23].

Prosser noted that the intrusion in the first privacy right had expanded beyond physical intrusion, and pointed out that Warren and Brandeis had been concerned primarily with the second privacy right. Nevertheless, Prosser felt that both real abuses and public demand had led to general acceptance of these 4 types of privacy invasions. Thomas Nagel, one of the America’s top contemporary philosophers, gives a more contemporary (philosophical) discussion of privacy, concealment, publicity, and exposure [25].

More recently, Adam Moore [26], building on the views of Ruth Gavison [27], Anita Allen [28], Sissela Bok [29], and others offered a control-over-access account of privacy. According to Moore, privacy is a cultural- and species-relative right to a level of control over access to bodies or places of information. While defending the view that privacy is relative to species and culture, Moore argues that privacy is objectively valuable: human beings that do not obtain a certain level of control over access will suffer in various ways. Moore claims that privacy, like education, health, and maintaining social relationships, is an essential part of human flourishing or well-being [23].

In a medical context, as viewed by Allen [13], the privacy at issue is very often confidentiality [30], specifically the confidentiality of patient-provider encounters (including the fact that an encounter has taken place), along with the secrecy and security of information memorialized in physical, electronic, and graphic records created as a consequence of these encounters [30]. Confidentiality is defined as restricting information to persons belonging to a set of specifically authorized recipients [13,28,31,32]. Confidentiality can be achieved through either professional silence, leaning on the moral aspect, or through secure data management [33], leaning on technologies and techniques.

The moral significance attached to medical privacy is reflected in data protection and security laws adopted by local and national authorities around the world. The point of these laws is to regulate the collection, quality, storing, sharing, and retention of health data, including the EHR [13].

### Medical Data Legal Regulations in the United States and Europe

#### United States

In the United States, several prominent cases in the 1990s aroused public and legal interest in privacy and confidentiality of medical data. There was no federal law regulating privacy and confidentiality before 1996. One of the key turning points was a breach of Nydia Velasquez’s medical records during her campaign for a House seat. At hearings before the US Senate Subcommittee on Technology and the Law of the Committee on the Judiciary on January 27, 1994, she said:

...I woke up one morning with a phone call from my friend Pete Hamill, a columnist at the New York Post. He told me that the night before, the Post had received an anonymous fax of my records from St. Claire Hospital. The records showed that I had been admitted to the hospital a year ago seeking medical assistance for a suicide attempt. He told me that other newspapers across the city had received the same information, and the New York Post was going to run a front page story the next day. For the press, it was a big story. For me, it was a humiliating experience over which I had no control...When I found out that this information was being published in the newspaper and that I had no power to stop it, I felt violated. I trusted the system and it failed me. What is most distressing is that once medical records leave the doctor’s office, there are no Federal protections to guard against the release of that information. In some States, it is easier to access a person’s medical history than it is to obtain the records of a person’s video rentals... [34]

Many similar stories have urged US legislators to adopt federal regulations implemented under the Health Insurance Portability and Accountability Act (HIPAA) of 1996 [35]. Before the HIPAA, no generally accepted set of security standards or general requirements for protecting health information existed in the health care industry. Under HIPPA, the US Department of Health and Human Services (HHS) has adopted 5 administrative rules, among them the HIPAA Privacy Rule [36] and the HIPAA Security Rule [37], the latter complementing the former. The Privacy Rule deals with all protected health information (PHI) regardless of the form (ie, including paper and electronic formats), and the Security Rule deals specifically with electronic PHI (ePHI).

The HIPAA Privacy Rule, or the *Standards for Privacy of Individually Identifiable Health Information*, is a set of federal standards to establish protection of certain health information. The *Security Standards for the Protection of Electronic Protected Health Information* (the Security Rule) established a national set of security standards for protecting certain health information that is held or transferred in electronic form. The Security Rule operationalizes the protections contained in the Privacy Rule by addressing the technical and nontechnical safeguards that organizations, called *covered entities*, must put in place to secure individuals’ ePHI. [38]. The Security Rule specifies administrative, technical, and physical measures that must be adopted by covered entities to adequately protect the privacy and confidentiality of ePHI.

Additionally, the HHS issued a set of rules [39] requiring the covered entities to notify individuals when their health information is breached. Furthermore, the covered entity must inform the HHS Secretary and the media when a breach involves more than 500 persons; thus, implementing provisions of the Health Information Technology for Economic and Clinical Health (HITECH) Act. The rules also apply to the business associates of the covered entities to notify the covered entity of events that affect privacy and confidentiality of ePHI at or by the business associate.

In the Breach Notification Rule [39], the HHS has specified the encryption and destruction as the technologies and methodologies that render PHI unusable, unreadable, or indecipherable to unauthorized individuals. Entities subject to the HHS and Federal Trade Commission regulations that secure health information as specified by the guidance through encryption or destruction are relieved from having to notify in the event of a breach of such information [39,40].

#### Europe

In 1995, the European Parliament passed Directive 95/46/EC on the protection of individuals in regard to the processing of personal data and the free movement of such data [41]. Member States in the European Union can, within the limits of the provisions of the Directive, determine more precisely the conditions under which the processing of personal data is lawful. Based on the Directive, the European Parliament and the Council on December 18, 2000, adopted the Regulation (EC) No 45/2001 on the protection of individuals in regard to the processing of personal data by the Community institutions and bodies and the free movement of such data [42].

Interestingly, Article 8 of the Directive 95/46/EC explicitly prohibits the processing of special categories of data, including the processing of data concerning health. However, the prohibition does not apply where processing of the data is required for the purposes of preventive medicine, medical diagnosis, the provision of care or treatment, or the management of health care services, and where those data are processed by a health professional subject under national law or rules established by national competent bodies to the obligation of professional secrecy or by another person also subject to an equivalent obligation of secrecy [41].

Furthermore, Article 17 of the directive prescribes security of processing. The controller of data must implement appropriate technical and organizational measures to protect personal data against accidental or unlawful destruction or accidental loss, alteration, or unauthorized disclosure or access, in particular where the processing involves the transmission of data over a network, and against all other unlawful forms of processing. Having regard to the state of the art and the cost of their implementation, such measures shall ensure a level of security appropriate to the risks represented by the processing and the nature of the data to be protected. The controller must, when processing is carried out on his behalf, choose a processor providing sufficient guarantees in respect of the technical security measures and organizational measures governing the processing to be carried out, and must ensure compliance with those measures. Processing by way of a processor must be governed by a contract or legal act binding the processor to the controller, stipulating that (1) the processor shall act only on instructions from the controller, and (2) the obligations regarding the appropriate technical and organizational measures to protect personal data, as defined by the law of the Member State in which the processor is established, shall also be incumbent on the processor. The contract or the legal act between the controller and the processor relating to data protection and the appropriate technical and organizational measures to protect personal data must be in written form [41].

When personal data are processed by automated means, measures shall be taken as appropriate in view of the risks. The measures should ensure that during communication of personal data and during transport of storage media, the data cannot be read, copied, or erased without authorization [42].

Directive 95/46/EC has been unchanged in principle since 1995. At the beginning of 2012, the European Commission proposed a comprehensive reform of the 1995 data protection rules to strengthen online privacy rights and boost Europe’s digital economy. Technological progress and globalization have profoundly changed the way the data are collected, accessed, and used. In addition, the 27 EU Member States have implemented the 1995 rules differently, resulting in divergences in enforcement. The proposed law would restrict the way Internet companies can gather, use, and retain the volumes of personal data that their users post online [43]. Among other measures, the use of encryption standards may be required in certain situations (Article 27), and a 24-hour notification rule is proposed: in a case of a personal data breach, the controller must notify, without undue delay and, when feasible, not later than 24 hours after having become aware of it, the personal data breach to the supervisory authority (Article 28). The European regulation, once passed, could serve as a template for other countries as they draft or revise their data protection policies [44], and it is threatening the current business practices of the Internet giants, such as Facebook [45].

#### Technological Similarities in Protecting Medical Data in the United States and Europe

The main difference between the American and European legislation pertaining to medical data is in the level of detail of how the data should be protected. The HIPAA and the accompanying rules, especially the Privacy and Security Rules, give great detail in how to protect data. In Europe, the detail of the protection is left to the EU Member States who must apply national provisions pursuant to Directive 95/46/EC within 3 years from the adoption of the directive.

However, there is one common point: both systems suggest the use of encryption to protect sensitive data. Although the HIPAA Security Rule does not dictate the use of encryption, it becomes an evident choice when considering the HITECH Breach Notification Rule. Entities covered by the rule are relieved from having to notify the media and others in the event of a breach of encrypted information. The EU Regulation (EC) No 45/2001, based on the Directive 95/46/EC, suggests the use of encryption when processing data for historical, statistical, or scientific purposes (Article 4) [42]. On the other hand, local laws of EU Member States usually do not dictate the use of encryption, as in case of the Data Protection Act of 1998 in the United Kingdom [46]. The same, for example, is true for the German Federal Act on Protection of Data [47]. It seems that recommendations to use encryption are lowered to the level of various guidance and recommendations [48,49].

Regardless of the legal system and local rules, the use of encryption seems an obvious choice for protecting medical data.

### Technology to Increase Confidentiality and Privacy With Outsourced Data Analyses

The fact that the outsourced data analyses poses a potential security threat to data has been well known for decades [50]. To protect sensitive data, several techniques have been developed.

Firstly, the techniques developed for the protection of statistical databases can be used. The goal of these techniques is to disclose the statistical data (eg, sums, counts, averages, minimums, maximums) without exposing sensitive individual records [51]. In these cases, the sensitive individual data values are either generalized or not disclosed. In the data analysis world, we cannot have data that have been generalized or are not available at all.

A typical result of an intelligent data analysis is a set of decision rules. A decision rule is a function which maps an observation to an appropriate action. Such rules are typically found in a medical diagnosis process in which several measurements are observed and an action is taken (eg, a drug is prescribed). For example, a computer-generated decision rule on generalized and not disclosed data would read: if a patient’s 2-hour postload plasma glucose level is ≤199 mg/dL and the patient’s body mass index (BMI) and age are unknown, then diagnosis of diabetes mellitus is negative. However, the American Diabetes Association recommends a postload glucose level ≤155 mg/dL with a 75 g glucose load [52]. High values may indicate diabetes and the doctors will not use just a single test result (measurement) to diagnose diabetes mellitus. If a doctor receives nondisclosed data (or a rule created on nondisclosed data) from the computer-assisted decision system, she has no use of it because additional data are needed for the final decision.

Secondly, one can modify the real value of an attribute using a value-class membership technique or value distortion [53] and try to reconstruct the original distribution as close as possible [54]. In the first case, the values are partitioned into a set of disjointed, mutually exclusive classes; for example, the numeric value of 2-hour postload glucose can be divided into 3 separate disjointed classes (c), 0-139 mg/dL, 140-199 mg/dL, and 200-299 mg/L, written as c_1_ = (0..139), c_2_ = (140..199), and c_3_ = (200..999), respectively. The selection of classes needs to be done carefully based on the domain knowledge; otherwise, the approach is useless. In the second case, the values are slightly changed, namely a random value drawn from some distribution is added to the original value. This approach can be used for numeric attributes (only) and for constructing a classifier [53]. Previous research focused on cases in which the data were distorted, expecting that the data were (deliberately) changed at the entry point into a system. In many cases, the models built were very sensitive to distorted values. For example, a computer-generated decision rule on distorted data may read: if the 2-hour postload plasma glucose level is ≤150 mg/dL and the BMI is >35 kg/m^2^ and age is ≤35 years, then diabetes mellitus diagnosis is negative, instead of the original if the 2-hour postload plasma glucose level is ≤127 mg/dL and BMI >26.4 kg/m^2^ and age is ≤28 years, then diabetes mellitus diagnosis is negative. An action based on a wrong decision rule can have serious consequences, especially in cases in which the values are very sensitive to small changes. However, the mentioned works are orthogonal to the work presented in this paper and can be used complementarily, if needed.

Thirdly, the encryption techniques can be implemented so that the data are encrypted on-site before they are sent for analysis. An analyst decrypts the data based on a password that was previously agreed upon and works with the original values. Typically, a data owner stores the data in an Excel or Word file and protects it by using the internal protection methods; alternatively, the data are stored in another format and compressed using WinZip tools, again protecting it with an internal protection method. The files are then transported to the outside world. Such a procedure has many drawbacks. Firstly, the data are not protected once the outsourced external analyst receives the files and deactivates the files’ internal protection to access the data. The data are vulnerable to any and all attacks possible once residing on the analyst’s computer. Secondly, the password with which the files are protected can easily be broken. A recent study showed that 93% of test files containing sensitive medical data could be recovered within a 24-hour period by using commercially available tools [55]. Interestingly, nothing has changed in the terms of using strong passwords for decades [56,57]. It can be concluded that passwords will continue to be the weakness of computing security.

### The Contribution

The aim of the present work is to develop a procedure to protect medical data in such a way that the outsourced analyst is capable of doing analyses on protected data and the results will be comparable, if not the same, as if they had been done on the original data by following the PbD principle. We tested this hypothesis by determining whether there were differences between outsourced decision trees built on encrypted data and the ones built on original data.

## Methods

### Formal Setting for Encrypting Data for Outsourced Analyses

In our proposed method, we avoided the weaknesses of the previously mentioned approaches. The data values were encrypted in such a way that outsourced data analyses were still possible, but the data remain encrypted and protected. This can be done by using a strong encryption algorithm, such as those approved by National Institute of Standards and Technology (NIST): Triple Data Encryption Algorithm (TDEA) [58], Advanced Encryption Standard (AES) [59], or Skipjack [60], so that the security should rely only on secrecy of the keys [61].

The formalization of the approach is presented in Multimedia Appendix 1 and is based on the flat file format (in principle, a textual file with data items separated by a comma), which is the usual format for data analytic tools [62].

### The Algorithm for Protection of Data for Decision-Making Analyses

We designed an algorithm that encrypts a flat file with plain text data into an encrypted flat file in such a way that external data analyses are still possible. The algorithm is presented in Multimedia Appendix 2.

For clarity of the proposed approach, let us take a closer look at an experiment with real-world examples from the medical and life sciences fields.

### Data Collection

For the purpose of demonstrating the usability of the proposed approach, we used all publicly available datasets from the University of California at Irvine (UCI) Machine Learning Repository [63], with the following restriction: the problem task was classification, data type was multivariate, from the life sciences area, and the data were in matrix (table) format. The UCI Machine Learning Repository lists 41 such datasets [64]. We further removed the following 11 datasets: Arcene, Dorothea, and p53 Mutants (the number of attributes >1000, the primary task is feature selection, not classification), both of the Kyoto Encyclopedia of Genes and Genomes (KEGG) datasets and the PubChem Bioassay Database (textual data), Parkinson’s (time series data), and the Thyroid Disease family of datasets (the task is from domain theory). Next, we used only original or larger datasets in which several sub-datasets were available (removed Breast Cancer Wisconsin Diagnostic and Prognostic, Soybean-small, SPECT Heart). We ended up experimenting with 30 datasets.

Most of the datasets in Attribute-Relation File Format (ARFF) were taken from the Software Environment for the Advancement of Scholarly Research (SEASR) repository [65], the rest were converted to ARFF from the UCI repository files by the authors. The original ARFF files are included in Multimedia Appendix 3.

### Data Processing

For the analytics tool in this experiment, we chose the J48 decision tree builder with standard built-in settings and initial values, which is freely available from the Waikato Environment for Knowledge Analysis (Weka) project toolkit [62] version 3.6.8. J48 is java-based decision tree builder based on a Quinlan’s C4.5 tree induction [66].

**Figure 1:**
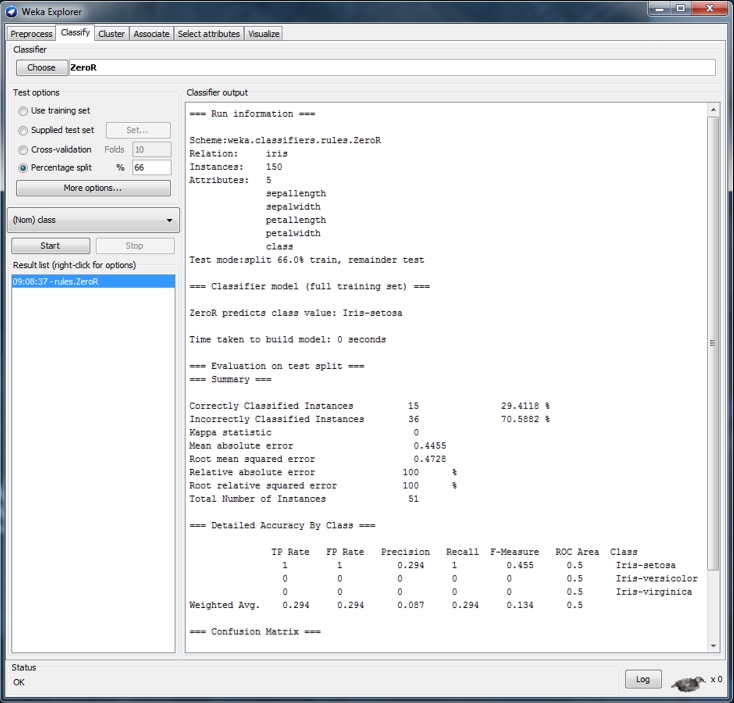
Weka Explorer user interface showing settings used.

First, each original dataset was used to build a decision tree using the J48 decision tree (see Figure 1). We used 66% of all dataset items for training and the remaining data were used for testing the model; therefore, we ignored any separate training or test set, or any associated cost model.

For each decision tree model, we measured the number of leaves, size of the tree, and the percentage of correctly classified instances (see Multimedia Appendix 4). The number of leaves defines the total number of decision rules included in a tree. The size of a tree gives the number of nodes (measurements) in a tree: the higher the number of nodes, the more complicated the rules are. The percentage of correctly classified instances (ie, accuracy) measures how many mistakes the computer-generated decision trees make when they are tested on real-world data. Measuring only the accuracy is not enough because many different trees based on different data can have identical accuracy.

Secondly, each data file was protected with the proposed algorithm (see Multimedia Appendix 5). We implemented a prototype with limited features in JavaScript language (see Multimedia Appendix 6). The advantage of using JavaScript is that the data are not sent to a server residing elsewhere, but are processed in a browser locally. We used the AES algorithm with 256-bit key on all string-, categorical-, or nominal-type attribute values. For numeric values, we simply multiplied the original values by 2 and added 1, thus hiding the original values. In real life situations, any numeric transformation preserving the desired statistical properties of data can be used [51]. Then, decision trees were built for each protected dataset with the same settings as the original datasets. Finally, the number of leaves, the size of the tree, and the percentage of correctly classified instances were measured again (see Multimedia Appendix 7).

### Hypotheses

For the approach to be useful there should be no statistically significant difference between the original and encrypted trees in terms of tree size, number of leaves in a tree, and the accuracy of the tree. Our hypotheses were (1) the mean tree size after encryption would be the same as before encryption, (2) the mean number of leaves after encryption would be the same as before encryption, and (3) the mean accuracy after encryption would be the same before encryption.

### Statistical Analysis

The same subject (a decision tree built with a specific dataset) was observed under 2 different conditions. The “before” samples were made of decision trees built on original data, and the “after” samples were made of decision trees built on encrypted data. Bootstrapped paired *t* tests for 2 dependent samples were used to identify whether significant differences occurred because of encryption of data on 3 independent variables: tree size, number of leaves, and accuracy. We considered differences to be significant at the <.05 level. SPSS version 21 (IBM Corp, Armonk, NY, USA) was used for analysis.

## Results

### Differences Between Decision Trees Based on Original and Protected Data

First, we tested if the decision trees built on original and protected data were different. Table 1 lists the size of a tree, the number of leaves in a tree, and the percentage of correctly classified items for each dataset when the tree was built on original data and when it was built on the protected data.

**Table 1:** Results of analyses on original and encrypted data files for tree size, number of leaves, and accuracy.

Database name	Original dataset			Encrypted dataset		
	Tree size^a^, n	Leaves^b^, n	Accuracy^c^, %	Tree size^a^, n	Leaves^b^, n	Accuracy^d^, %
Abalone	2312	1183	21.97	2312	1183	21.97
Acute inflammations	5	3	100.00	5	3	100.00
Arrhythmia	99	50	71.43	99	50	71.43
Audiology (standardized)	54	32	83.12	54	32	83.12
Breast cancer	6	4	68.04	6	4	68.04
Breast cancer Wisconsin (original)	27	14	95.38	27	14	95.38
Breast tissue	29	15	47.22	29	15	47.22
Cardiotocography	19	14	98.34	33	25	98.34
Contraceptive method choice	263	157	55.29	263	157	55.29
Covertype	29,793	14,897	93.59	29,793	14,897	93.59
Dermatology	41	31	92.74	41	31	92.74
Echocardiogram	9	5	70.37	9	5	70.37
Ecoli	43	22	78.95	43	22	78.95
Haberman’s survival	5	3	75.96	5	3	75.96
Hepatitis	21	11	79.25	21	11	79.25
Horse colic	29	18	68.55	29	18	68.55
Iris	9	5	96.08	9	5	96.08
Lung cancer	16	10	63.64	16	10	63.64
Lymphography	34	21	78.00	34	21	78.00
Mammographic mass	15	12	82.26	15	12	82.26
Mushroom	30	25	100.00	30	25	100.00
Pima Indians diabetes	39	20	76.25	39	20	76.25
Post-operative patient	1	1	70.97	1	1	70.97
Primary tumor	88	47	39.13	88	47	39.13
Seeds	15	8	97.18	15	8	97.18
Soybean (large)	93	61	90.52	93	61	90.52
Spectf heart	17	9	66.67	17	9	66.67
Statlog (heart)	45	27	76.09	45	27	76.09
Yeast	369	185	58.81	369	185	58.81
Zoo	17	9	94.12	17	9	94.12

^a^Number of nodes (measurements) in a tree.

^b^Number of decision rules in a tree.

^c^Percentage of correctly classified original items with respect to all items (ie, the number of times the tree's rules lead to the right decision).

^d^Percentage of correctly classified encrypted items with respect to all items (ie, the number of times the tree's rules lead to the right decision).

The analysis of the results showed that all but 1 of the encrypted decision trees were identical to the original ones on all 3 attributes: tree size, number of leaves, and accuracy. The only difference was with the tree built on the Cardiotocography dataset, in which the size of the tree and the number of leaves were different (tree size: 19 vs 33; leaves: 14 vs 25 for original and encrypted datasets, respectively). The difference is due to internals of the algorithm building a decision tree: the algorithm decides how to build the decision tree based on the measurement values; when they are the same, the decision how to build is based on the measurement names, which are not preserved with encryption. Because the values are the same, the induced decision trees are different only in the structure and not in the accuracy or the meaning of the rules.

We tested our hypotheses by using bootstrapped paired samples *t* tests, (see Multimedia Appendix 8). The paired samples results are shown in Table 2.

The unusually high standard deviation indicates the presence of outliers in the data. The outliers are in the Abalone and Covtype data. Outliers tend to increase the estimate of sample variance; thus, decreasing the calculated *t* statistic and lowering the chance of rejecting the null hypothesis. Therefore, we used bootstrapping for the paired samples test, which makes no assumption about underlying population distributions [67]. The results of the bootstrapped paired samples tests are presented in Table 3.

With a significance of *P*=.19, we cannot reject the hypotheses that the mean difference in tree size and in number of leaves would be the same as before encryption. The before and after samples are the same, so we retain the hypothesis that the mean accuracy after encryption would be the same as before encryption.

**Table 2:** Paired samples statistics.

Pairs		Mean	SD	SEM
**Pair 1, n=30**				
	Original size	1118.1	5432.1	991.8
	Encrypted size	1118.6	5432.0	991.8
**Pair 2, n=30**				
	Original leaves	563.5	2715.7	495.8
	Encrypted leaves	563.7	2715.7	495.8
**Pair 3, n=30**				
	Original accuracy	0.763^a^	0.189	0.034
	Encrypted accuracy	0.763^a^	0.189	0.034

^a^The correlation and *t* test cannot be computed because the standard error of the difference is zero.

**Table 3:** Bootstrapped paired samples test results.

Pairs	Mean	Bias	SEM	95% CI	*P*
Pair 1: Original size–encrypted size	–0.5	–0.3	0.4	–2.1, –0.5	.19
Pair 2: Original leaves–encrypted leaves	–0.2	–0.1	0.2	–0.9, –0.2	.19

### Usability of Encrypted Decision Trees

Secondly, we tested whether a decision tree built on encrypted data could be of any use to the data owner and how to make use of it. We will demonstrate the approach with the Pima Indian Diabetes Dataset [68,69]. This dataset has 768 instances and 8 attributes (columns or measurements that describe each instance): number of times pregnant (preg); plasma glucose concentration after 2 hours in an oral glucose tolerance test in mg/dL (plas); diastolic blood pressure in mm Hg (pres); triceps skin fold thickness in mm (skin); 2-hour serum insulin in μU/mL (insu); BMI in kg/m^2^ (mass); diabetes pedigree function (pedi); and age in years (age). The final prediction class, actually a rule based on measurements, was tested negative for diabetes (tested_negative) or tested positive for diabetes (tested_positive).

**Figure 2:**
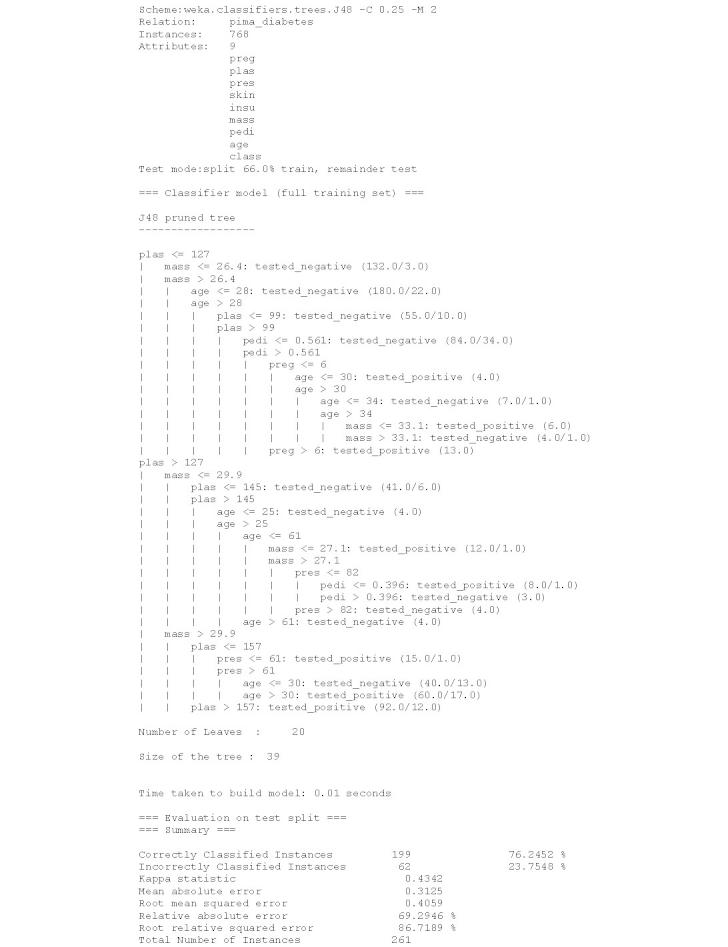
Decision tree model to assist diagnosing diabetes mellitus built with plain text data from the Pima Indians Diabetes Dataset.

**Figure 3:**
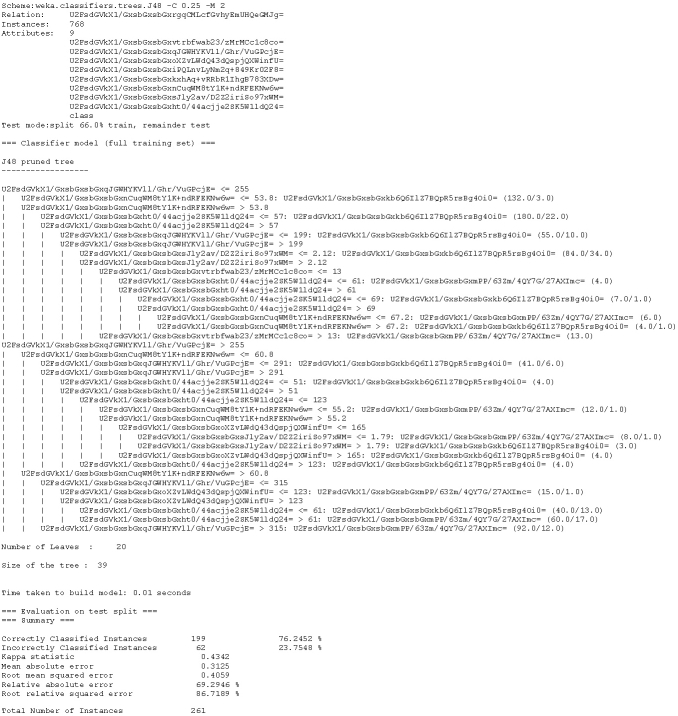
Decision tree model to assist in diagnosing diabetes mellitus built with encrypted data.

Based on the data, an external analyst (a medical expert) should be able to construct a decision tree that would be able to assist in diagnosing diabetes mellitus for each individual represented by data values in a record (tuple). The decision tree constructed from the original plain text dataset is depicted in Figure 2. The same tree built on encrypted data is depicted in Figure 3.

The trees are identical, except for the attribute names and values, which are encrypted. For example, if the data owner would like to decrypt the encrypted decision rule (see Multimedia Appendix 2), which would read “if [encrypted data]≤255 and [encrypted data]>53.8 and [encrypted data]≤57 then [encrypted answer]”, as seen in lines 1, 3, and 4 from the pruned decision tree, he or she would simply query the lookup table using Structured Query Language (SQL) or any SQL-based graphical tool [70]. The list of queries and the results are shown in Table 4.

Thus, the final decision rule, which was previously encrypted, now reads: IF 2_hr_postload_plasma_glucose <= 127 ∧ body_mass_idx > 26.4 ∧ age <= 28 THEN tested_negative (if 2-hour postload plasma glucose level is ≤127 mg/dL and BMI >26.4 kg/m^2^ and age ≤28 years then predict negative diabetes diagnosis).

**Table 4:** List of queries for transforming encrypted data to original.

Query	Result
SELECT original_atribute FROM lookup_table WHERE renamed_attribute=” U2FsdGVkX1/GxsbGxsbGxqJGWHYKVll/Ghr/VuGPcjE=”	2_hr_postload_plasma_glucose
SELECT original_value FROM lookup_table WHERE encrypted_value=255 AND attribute_name=”2_hr_postload_plasma_glucose”	127
SELECT original_atribute FROM lookup_table WHERE renamed_attribute=” U2FsdGVkX1/GxsbGxsbGxnCuqWM8tY1K+ndRFEKNw6w=”	Body mass index
SELECT original_value FROM lookup_table WHERE encrypted_value=53.8 AND attribute_name=”Body mass index”	26.4
SELECT original_atribute FROM lookup_table WHERE renamed_attribute=” U2FsdGVkX1/GxsbGxsbGxht0/44acjje2SK5W1ldQ24=”	Age
SELECT original_value FROM lookup_table WHERE encrypted_value=57 AND attribute_name=”Age”	28
SELECT original_value FROM lookup_table WHERE encrypted_value=” U2FsdGVkX1/GxsbGxsbGxkb6Q6IlZ7BQpR5rsBg4Oi0=” AND attribute_name=”Class”	Tested_negative

## Discussion

### Principal Findings

This study evaluated the use of a proposed algorithm on 30 publicly available datasets from the UCI repository. The aim of the study was to assess whether analyses on properly protected and encrypted data are possible without a data analyst having access to the original plain text data, which may not always be available because of internal and external restrictions of health care provider institutions.

The analysis showed that the results of building decision trees on original and protected (encrypted) data using the proposed algorithm are virtually identical. The trees were not significantly different based on the bootstrapped *t* tests (*P*=.19).

A decision tree, built on protected data (and the data themselves), is useless for an adversary because all the data are encrypted. The data owner can query the original source data to transform the encrypted data back to readable plain text.

### Use Cases and Limitations

There are 3 scenarios or reasons why one might consider using our solution. First, a lack of knowledge and expertise within the health care institution may prevent data processing for decision-making analyses. Secondly, the available resources may not be adequate to perform the analyses. Thirdly, the adherence to an organization’s security policy (eg, based on a need-to-know basis) may not allow for the analyses to be performed on unprotected data. Let us discuss the scenarios in more detail.

The first scenario is when there is a lack of knowledge to do the actual data processing for decision-making purposes and knowledge discovery. In the future, in-house data scientists may be trained so that this becomes less of a practical issue in the business context, but it may remain in the health care environment where the core business is providing health care–related services, not data analyses.

The second scenario is about the lack of computing-related resources that may be available within the health care institution. Although the proposed solution is demonstrated using open-source tools and datasets, customized third-party tools may be needed, which would involve third-party specialists in decision making. When the reason to outsource is because of insufficient computing resources (eg, computing power or storage capacity), one may consider using cloud computing services. These services still need to address the privacy issues (eg, [71,72]), although it is feasible to scale the proposed approach to use multiprocessor power available in the cloud computing environment [73-75].

The third scenario addresses the restrictions imposed on data processing that might exist within the organization because of internal or external rules and regulations. A restriction about data processing could have been set by an internal security policy [76], such as when a security policy based on a need-to-know rule is enforced (eg, [77-79]). The need-to-know rule specifies that the access to sensitive (medical) data is allowed only to those who need to know these data to perform their jobs. Typically, only medical personnel (doctors, nurses) need to have access to specific records to perform their job. Data analysts do not need the access to the undisclosed and unprotected data to perform their job. Rather, they can perform their duties on encrypted data, as suggested by our approach.

All 3 scenarios indicate that the data owner wants to outsource the ability to process the data without actually giving the processor access to it. This may seem contradictory, but we have shown that our approach is feasible if the data are encrypted. In this case, the data processor does not have (nor does she need) the key to decrypt the data, so she does not have the access to the plain text data. The data are safe even when being processed by a third party, or internally when the security policy requires it to be.

Nevertheless, an important limitation should be highlighted. Namely, the plain text data need to be either in numeric or textual (categorical or strings) form. The approach does not support the data mining or decision making on purely textual data. For these to be supported, further work is needed, including the incorporation of a homomorphic encryption scheme [80]. Nonetheless, most of the existing decision-making tools use numeric or categorical data.

### Comparison With Prior Work

The approach developed within our study can be used in conjunction with approaches presented by Adam and Wortman [51], Agrawal and Aggarwal [54], or by Agrawal and Srikant [53]. The approaches developed or presented by these authors aim toward blurring or not disclosing the original data. Their approach would produce slightly different analysis results if the original data were used. Our approach can, nevertheless, be applied after one of the previously developed approaches to fully prevent reconstructing the original data by means of statistical disclosure mechanisms.

The proposed solution follows the 7 principles [81] of the PbD [9-11] framework:

1. Proactive not reactive, preventive not remedial: Data are preventively encrypted so any disclosure has no intermediate consequences.
2. Privacy as the default setting: The maximum degree of privacy is delivered by ensuring that personal data are automatically protected in any given IT system or business practice.
3. Privacy embedded into design: Privacy is embedded into the design and architecture of health care IT systems and business practices.
4. Full functionality-positive-sum, not zero-sum: Accommodate all legitimate interests and objectives in a positive-sum win–win manner, not through a dated, zero-sum approach, where unnecessary trade-offs are made. The pretense of false dichotomies, such as privacy vs security, is avoided demonstrating that it is possible to have both.
5. End-to-end security-full lifecycle protection: By having the encryption embedded into the system before the first element of information is stored, the protection is extended throughout the entire lifecycle of the data involved, including during processing.
6. Visibility and transparency (keep it open): The component parts and operations, as proposed by our approach, remain visible and transparent to users and providers alike.
7. Respect for user privacy (keep it user-centric): By using the approach, the architects and operators are required to keep the interests of the individual uppermost by offering such measures as strong privacy defaults.

### Conclusions

Medical data stored in online systems are true goldmines. However, if they are not analyzed, they are useless. The problem of data analyses within health care organizations is that these organizations’ primary focus is providing health care services and they rarely have enough computing and employee resources to do the analyses. The obvious choice is to use external third-party analysis services. However, exporting sensitive medical data to the outside world can be exposed to significant risks and keeping the medical data safe within health care organizations is also an organizational and technological challenge. Being responsible for someone else’s potential mistakes can easily tip the decision toward not using external analyses. Because of time constraints, many health care goals, and the tasks or decisions needed to pursue those goals, these are intentionally deferred until a future opportunity [82], if it ever comes.

It was observed that traditional EHRs have no security guarantee outside the health care service domain [15,16]. The technology that can help is available: the proposed algorithm can be considered as an interface between a data owner from the health care service domain on one side and an outside data analyst on the other side. The design of the algorithm is such that the data are protected in a manner that data analyses are still possible, yet not decipherable by a third party at the same time. Thus, the algorithm conforms to the strict regulations regarding the use and processing of medical data, such as the HIPAA rules and the EU’s Directive 95/46/EC. Any potential breach that would involve the data protected with the proposed algorithm is exempt from the HITECH Breach Notification Rule.

In our study, we investigated the feasibility of using encryption within the decision-making process. We tested our approach on 30 databases only. As a part of our future work, further studies with different databases and different types of decisions will be performed to confirm this study’s results.

The results of our research confirm that data analyses conducted on protected data can be equivalent to those on original unprotected data. This study’s results are promising and provide evidence that the method works. However, more study is needed to show that the method works in all cases.

The procedure can be fully automated. The data owner and the data analyst can seamlessly exchange the data and the results. Importantly, the data and the results are safe while in transit and during processing with the data analyst. The data analyst is not required to implement any additional security measures because these were already implemented at the data owner’s side. The proposed approach is compatible with all 7 foundational principles of the PbD framework. By following the PbD framework, we can harness large amounts of data to gain valuable insights into the health system and the health of populations to improve clinical outcomes and achieve cost efficiencies without intruding on privacy [83].

## Multimedia Appendix 1

Formal description of data-protecting algorithm.

## Multimedia Appendix 2

The algorithm for protection of data for decision-making analyses.

## Multimedia Appendix 3

Datasets from UCI Repository in original UCI format and/or ARFF format.

## Multimedia Appendix 4

Decision trees built on original data.

## Multimedia Appendix 5

Datasets from UCI Repository in ARFF format, protected with the proposed algorithm.

## Multimedia Appendix 6

The proposed algorithm prototype with limited functionality implemented in JavaScript.

## Multimedia Appendix 7

Decision trees built on protected data.

## Multimedia Appendix 8

SPSS files with result of bootstrapped two dependent samples paired *t* test.

## Abbreviations

AES: Advanced Encryption Standard
ARFF: Attribute-Relation File Format
EHR: electronic health records
ePHI: electronic protected health information
HHS: US Department of Health and Human Services
HIPAA: Health Insurance Portability and Accountability Act of 1996
HITECH: Health Information Technology for Economic and Clinical Health Act
KEGG: Kyoto Encyclopedia of Genes and Genomes
NIST: National Institute of Standards and Technology
PbD: Privacy by Design
PHI: protected health information
SEASR: Software Environment for the Advancement of Scholarly Research
SQL: Structured Query Language
TDEA: Triple Data Encryption Algorithm
UCI: University of California at Irvine
