# Supplementary material for: Outsourcing Medical Data Analyses: Can Technology Overcome Legal, Privacy, and Confidentiality Issues?
Source: J Med Internet Res. 2013 Dec 16;15(12):e283. doi: 10.2196/jmir.2471 (PMC3877744; doi:10.2196/jmir.2471)
Supplement: Supplementary file 1 [file jmir_v15i12e283_app1.pdf]

Multimedia Appendix 1: Formal Description of Data-protecting Algorithm

Suppose we have the original data stored in a flat file FF, which is a usual format for data analytic tools [1]. A flat file is representing a single table T. A table is consisting of a *table scheme* T, which is invariant over the life of the table, and a set of *tuples* consistent with the scheme. Such a set of tuples is called the current *value* or *instance* of table T. A table schema of T, TS, is described by a finite set of *attributes*, $TS = \{A_1, A_2, \dots, A_N\}$. Each attribute A_i is the name of a role played by some domain D_i in the relation schema TS. We assume that any attribute name A_i appears only once within table schema TS. D_i is called the domain of A_i and is denoted by $D_i = dom(A_i)$. A *domain* is simply a set of values and can be finite or infinite. In a relational table, we have a set of domains, $D = \{D_1, \dots, D_k, \dots, D_K\}$. A *table* T consists of a table scheme TS and a set of N-tuples t, $t = \{t_1, \dots, t_m, \dots, t_M\}$. Each N-tuple t_m (belonging to the search table T) is an ordered list of N values, $t_m = \langle v_{m1}, \dots, v_{mn}, \dots, v_{mN} \rangle$, where each value v_{mn} is an element of $dom(A_n)$, or is a special *null* value. The nth value (v_{mn}) of the mth tuple (t_m) of search table T, which corresponds to the attribute A_n, is referred to as v_{mn}=$t_m[A_n]$.

The data-protecting algorithm is described formally as follows.

Definition 1: Let D_n and $D_n^{\#}$ be sets. A *function* from D_n into $D_n^{\#}$ is a subset F of $D_n \times D_n^{\#}$ such that for each element $a \in D_n$ there is exactly one element $b \in D_n^{\#}$ such that $(a, b) \in F$. Set D_n corresponds to domains of relation schema TS.

Definition 2: Let $D^{\#}$ be a set of domains, such that $D^{\#} = \{D_n^{\#} \| D_n^{\#}| = |D_n|\}$. Let $D_n \to D_n^{\#}$ be a function, and $F^D = \{f_n()\}$ be a set of transformations f_n. Transformation f_n is said to transform D_n onto $D_n^{\#}$, if

1. $\forall b \exists a: f_n(a) = b$
2. $f_n(a_1) = f_n(a_2) \Leftrightarrow a_1 = a_2$

In definition 2 we have defined a set of functions that encrypt the original values to encrypted ones. If a value comes from a categorical domain, the function f_i is simply a strong encryption algorithm, e.g. those approved by National Institute of Standards and Technology (NIST): TDEA (3DES) [2], AES [3], or Skipjack [4]. If a value is numeric, the function needs to be chosen such that it scrambles the original numbers and that it preserves statistical properties of data [5].

Definition 3: Let RN: *String* → *String* be a function such that

1. $\forall b \exists a: RN(a) = b$
2. $RN(a_1) = RN(a_2) \Leftrightarrow a_1 = a_2$

This way we defined a function that transforms attribute names into new (encrypted) values. The function RN is a rename operation.

The functions RN and F^D, together with corresponding keys, are secret, known only by the data owner; the attacker might guess the functions so the security should rely only on secrecy of the keys [6].

References

1. Witten IH and Frank E. Data Mining: Practical machine learning tools and techniques. 2nd ed. Morgan Kaufmann; 2005. ISBN: 0120884070
2. NIST. Recommendation for the Triple Data Encryption Algorithm (TDEA) Block Cipher. Springfield, VA, USA: Federal Information Processing Standards Special Publication 800-67 (FIPS PUB 800-67); 2012.
3. NIST. Advanced Encryption Standard (AES). Springfield, VA, USA: Federal Information Processing Standards Publication 197 (FIPS PUB 197); 2001.

4. NIST. Escrowed Encryption Standard (EES); SKIPJACK and KEA Algorithm Specifications. Springfield, VA, USA: Federal Information Processing Standards Publication 185 (FIPS PUB 185); 1998.
5. Adam NR and Wortmann JC. Security-control methods for statistical databases: a comparative study. ACM Comput Surv 1989; 21(4): 515-556. DOI: 10.1145/76894.76895.
6. Stallings W. Cryptography and Network Security, Principles and Practices. 4th ed. Prentice Hall; 2006. ISBN: 0131873164
