# Supplementary material for: Outsourcing Medical Data Analyses: Can Technology Overcome Legal, Privacy, and Confidentiality Issues?
Source: J Med Internet Res. 2013 Dec 16;15(12):e283. doi: 10.2196/jmir.2471 (PMC3877744; doi:10.2196/jmir.2471)
Supplement: Supplementary file 2 [file jmir_v15i12e283_app2.pdf]

Multimedia Appendix 2: The Algorithm for Protection of Data for Decision-Making Analyses

We designed an algorithm which encrypts a flat file FF with plaintext data into a flat file FF#, containing encrypted and protected data in such a way that the external data analyses are still possible. The flat file FF# can be exported to the outside world without a fear that the contents would be exposed.

Algorithm A:

```
STEP 01: OPEN (FF);                          //  open a flat file containing plaintext data
STEP 02: CREATE (FF#=RN(FF));                //  create a new file (FF#) with encrypted data,
                                                 renamed
STEP 03: CREATE (LT);                        //  create a lookup table for future decryption
STEP 04: WHILE attribute_definition DO       //  at the beginning of the FF there are
                                                 attribute definitions
STEP 05:   READ (FF, An, At);                //  read the attribute name and its domain type
STEP 06:   WRITE (LT, An, RN(An));           //  write into a lookup table the original and
                                                 encrypted attribute name
STEP 07:   WRITE (FF#, RN(An));              //  write the renamed (encrypted) attribute to
                                                 the encrypted file
STEP 08: WHILE NOT END_OF_FILE (FF) DO       //  go into a loop in which each data line
                                                 (tuple) will be encrypted and stored to a
                                                 new file
STEP 09:   READ (FF, data_m);                //  read a m^th tuple from plaintext file
STEP 10:   WITH EACH A_n DO                  //  for each m^th tuple (data line) go into a loop
                                                 for each attribute A_n
STEP 11:     v#_mn := f_n(data_m[A_n]);      //  encrypt each data element with its own
                                                 function f_n
STEP 12:     WRITE (LT, data_m[A_n], v#_mn, An);  //  Store original and encrypted value plus the
                                                 corresponding attribute name into a lookup
                                                 table
STEP 13:     data# := data# || v#_mn;        //  add each encrypted value of an attribute A_n
                                                 to encrypted data line
STEP 14:   WRITE (FF#, data#);               //  write encrypted data line into an encrypted
                                                 file
STEP 15: CLOSE (FF); CLOSE (FF#); CLOSE (LT);  //  close files
```

▪

The data can be protected by using the above algorithm. The resulting FF# file can be sent for the analyses without a fear of the values ever being exposed to a third (unauthorized) party. A data owner retains the lookup table in which the corresponding plaintext and encrypted values are stored for future reference and for decryption of the analyst's results. The data analyst would return the results to the data owner, e.g. in form of rules, such as

```
IF "U2FsdGVkX1/yqxl/eyk52z8mF8jB7h9f62FHgBroM1Y=" <= 279 ∧
"U2FsdGVkX19llZHZPTknyI85k3Zo6amgHaRFkihmRS8=" > 53.8 ∧
"U2FsdGVkX1+NUWjhelAQNQ+GOiQxIsIMJvHWSbfFDUY=" <= 57 THEN
"U2FsdGVkX19XzMvcNLU/5Tl3pIgdirFb9nZipeIVApE=".
```

The data owner would then use the look-up table to transform the results back to the readable form.
