# Supplementary material for: Outsourcing Medical Data Analyses: Can Technology Overcome Legal, Privacy, and Confidentiality Issues?
Source: J Med Internet Res. 2013 Dec 16;15(12):e283. doi: 10.2196/jmir.2471 (PMC3877744; doi:10.2196/jmir.2471)
Supplement: Supplementary file 6 [file jmir_v15i12e283_app6.zip › encrypt.htm]

|  |  |
| --- | --- |
|  |  |
| Text to encrypt: |  |
| Password: |  |
| Alfa-numerical Algorithm | AES |
| Numerical Algorithm | 2x+1 |
| Encrypted/Decrypted text: |  |
| Encrypt Decrypt | |
